# One Health Genomic Surveillance of Escherichia coli Demonstrates Distinct Lineages and Mobile Genetic Elements in Isolates from Humans versus Livestock

**DOI:** 10.1128/mBio.02693-18

**Published:** 2019-01-22

**Authors:** Catherine Ludden, Kathy E. Raven, Dorota Jamrozy, Theodore Gouliouris, Beth Blane, Francesc Coll, Marcus de Goffau, Plamena Naydenova, Carolyne Horner, Juan Hernandez-Garcia, Paul Wood, Nazreen Hadjirin, Milorad Radakovic, Nicholas M. Brown, Mark Holmes, Julian Parkhill, Sharon J. Peacock

**Affiliations:** a London School of Hygiene & Tropical Medicine, London, United Kingdom; b Wellcome Trust Sanger Institute, Wellcome Trust Genome Campus, Hinxton, Cambridge, United Kingdom; c Department of Medicine, University of Cambridge, Addenbrooke’s Hospital, Cambridge, United Kingdom; d Clinical Microbiology and Public Health Laboratory, Public Health England, Cambridge, United Kingdom; e Cambridge University Hospitals NHS Foundation Trust, Cambridge, United Kingdom; f British Society for Antimicrobial Chemotherapy, Birmingham, United Kingdom; g Department of Veterinary Medicine, University of Cambridge, Cambridge, United Kingdom; h Royal (Dick) School of Veterinary Studies, University of Edinburgh, Edinburgh, United Kingdom; Pasteur Institute

**Keywords:** ESBL, *Escherichia coli*, antimicrobial resistance, genomics, livestock

## Abstract

Livestock have been proposed as a reservoir for drug-resistant Escherichia coli that infect humans. We isolated and sequenced 431 E. coli isolates (including 155 extended-spectrum β-lactamase [ESBL]-producing isolates) from cross-sectional surveys of livestock farms and retail meat in the East of England. These were compared with the genomes of 1,517 E. coli bacteria associated with bloodstream infection in the United Kingdom. Phylogenetic core genome comparisons demonstrated that livestock and patient isolates were genetically distinct, suggesting that E. coli causing serious human infection had not directly originated from livestock. In contrast, we observed highly related isolates from the same animal species on different farms. Screening all 1,948 isolates for accessory genes encoding antibiotic resistance revealed 41 different genes present in variable proportions in human and livestock isolates. Overall, we identified a low prevalence of shared antimicrobial resistance genes between livestock and humans based on analysis of mobile genetic elements and long-read sequencing. We conclude that within the confines of our sampling framework, there was limited evidence that antimicrobial-resistant pathogens associated with serious human infection had originated from livestock in our region.

## Abstract (precis)

The increasing prevalence of E. coli bloodstream infections is a serious public health problem. We used genomic epidemiology in a One Health study conducted in the East of England to examine putative sources of E. coli associated with serious human disease. E. coli from 1,517 patients with bloodstream infections were compared with 431 isolates from livestock farms and meat. Livestock-associated and bloodstream isolates were genetically distinct populations based on core genome and accessory genome analyses. Identical antimicrobial resistance genes were found in livestock and human isolates, but there was limited overlap in the mobile elements carrying these genes. Within the limitations of sampling, our findings do not support the idea that E. coli causing invasive disease or their resistance genes are commonly acquired from livestock in our region.

## INTRODUCTION

Escherichia coli is a leading cause of infection in hospitals and the community (1, 2). The prevalence of E. coli bloodstream infections has shown a marked increase in Europe and the United States since the early 2000s (3–6). This has been associated with the emergence and global dissemination of E. coli that produce extended-spectrum β-lactamases (ESBL-E. coli). Such isolates are resistant to many penicillin and cephalosporin antibiotics (3, 5) and are associated with excess morbidity, mortality, longer hospital stay, and higher health care costs compared with infections caused by E. coli that are not ESBL producers (7–9). Successful therapy has been further challenged by the emergence of multidrug-resistant (MDR) E. coli with acquired resistance to the carbapenem drugs and more recently to colistin, a drug of last resort for multidrug-resistant infections (10, 11).

Tackling the rising trends in prevalence of MDR E. coli infections in humans requires an understanding of reservoirs and sources for human acquisition. Food-producing animals have been proposed as a source of ESBL-E. coli in humans based on comparison of bacterial genotypes using multilocus sequence typing (MLST) (12–14). This method lacks sufficient discrimination to generate robust phylogenetic comparisons of population genetics and does not capture information on accessory genome composition such as genes encoding drug resistance. Whole-genome sequencing overcomes both of these limitations, but there are limited published data on the use of this technique to address the transmission of antibiotic-resistant E. coli between livestock and humans (15). Here, we report the findings of a genomic epidemiological investigation of E. coli sourced from livestock, meat, and patients with bloodstream infections within a tightly defined geographical location.

## RESULTS

### Isolation of E. coli from livestock farms and retail meat.

**FIG 1:**
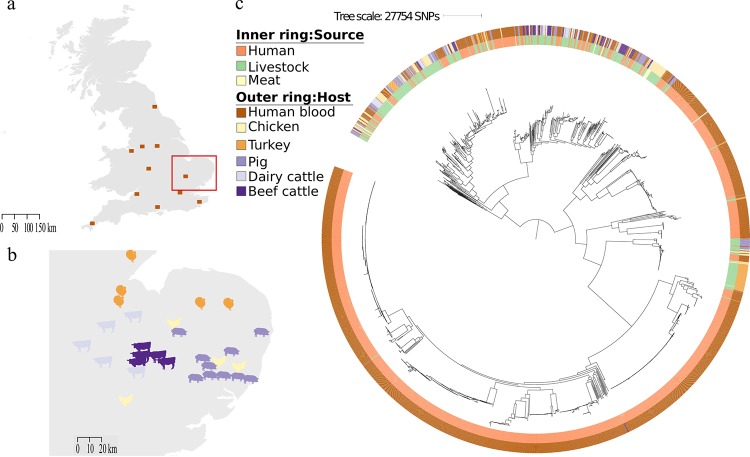
(a) Map of the United Kingdom showing the locations for the human clinical isolates, with the East of England highlighted by a red box. (b) Map of the East of England showing the locations of farms (images indicate livestock species). (c) Maximum likelihood tree based on SNPs in the core genes of 1,948 E. coli isolates cultured from livestock farms, retail meat, and patients with bloodstream infection.

A cross-sectional survey was performed between 2014 and 2015 to isolate ESBL-E. coli and non-ESBL-producing E. coli from livestock at 29 farms in the East of England, United Kingdom (UK) (10 cattle [5 beef cattle and 5 dairy cattle], 10 pig, and 9 poultry [4 chickens and 5 turkeys]) (Fig. 1b). A total of 136 pooled fecal samples (34 from cattle, 53 from pigs, and 49 from poultry) were collected and cultured, with an average of five pooled samples taken per farm. Numerous colonies were picked and identified from each sample to capture different lineages in the same sample. E. coli were isolated from all 29 farms, and ESBL-E. coli were isolated from 16 (55%) of these farms (see Table S1 in the supplemental material). The highest prevalence of ESBL-E. coli occurred in poultry farms (8/9), followed by pig farms (5/10) and cattle farms (3/10).

10.1128/mBio.02693-18.7 TABLE S1 Details of all *E. coli* isolates sequenced from livestock, meat, and human invasive infections. Download Table S1, XLSX file, 0.1 MB. Copyright © 2019 Ludden et al. 2019 Ludden et al. This content is distributed under the terms of the Creative Commons Attribution 4.0 International license.

A cross-sectional survey was performed in April 2015 to isolate ESBL-E. coli from 97 prepackaged fresh meat products purchased in 11 major supermarkets in Cambridge, East of England (5 to 16 products per supermarket) (16). These originated from 11 different countries, although the majority (69/97 [71%]) were from the UK. Nineteen ESBL-E. coli isolates were cultured from 17/97 (18%) products (2 isolates sequenced from 2 samples representing different antibiograms), of which 16 isolates were from chicken originating from the UK (*n* = 12), Ireland (*n* = 2), Hungary (packaged in Ireland; *n* = 1), or multiple origins (Brazil, Thailand, and Poland; *n* = 1), and 3 were from turkey (*n* = 2) and pork (*n* = 1) from the UK.

We sequenced a total of 431 E. coli isolates from livestock (*n* = 411) and meat (*n* = 20), of which 155 were ESBL-E. coli (136 from livestock and 19 from meat).

### Evaluation of an E. coli collection from patients with bloodstream infection.

A key study objective was to determine whether livestock and retail meat represented potential sources of E. coli associated with serious invasive disease in humans. In light of this, the human isolates used in the comparison with livestock isolates had caused bloodstream infection. A total of 1,517 open access E. coli genomes (142 ESBL and 1,375 non-ESBL) associated with bloodstream infections were retrieved (17, 18). Bloodstream isolates obtained from patients admitted to the Cambridge University Hospitals NHS Foundation Trust in the East of England between 2006 and 2012 (*n* = 424) (17, 18) were combined with bloodstream isolates submitted to the British Society of Antimicrobial Chemotherapy from 11 hospitals across England (*n* = 1,093) between 2001 and 2011 (locations shown in Fig. 1a, and full isolate listing in Table S1) (17, 18). A potential limitation of this human isolate collection is that they might overrepresent hospital-acquired isolates, while a comparison of E. coli from livestock would require a comparison of community-acquired bacteria. Two analyses were undertaken to evaluate this possibility. First, we defined where the bloodstream infection was acquired for 1,303 cases for whom we had this information. This demonstrated that 886/1,303 (66%) cases were community associated. We then constructed a maximum likelihood tree of the invasive disease genomes to compare the phylogeny of isolates associated with community- versus health care-associated disease (Fig. S1). This demonstrated that genomes from the two categories were intermixed and distributed across the phylogeny, with no evidence of clustering by origin of infection. We concluded that our invasive collection was likely to include strong representation of E. coli carried by people in the community.

10.1128/mBio.02693-18.1 FIG S1 Maximum likelihood phylogenetic tree constructed from core gene SNPs identified for 1517 *E. coli* isolates associated with bloodstream infection at 11 hospitals in the UK between 2001 and 2012. The tree is annotated by place of onset of infection. Download FIG S1, TIF file, 1.2 MB. Copyright © 2019 Ludden et al. 2019 Ludden et al. This content is distributed under the terms of the Creative Commons Attribution 4.0 International license.

### Genomic comparison of E. coli from patients with bloodstream infection, livestock, and retail meat.

We combined and compared the 1,517 human invasive *E*. *coli* genomes with the 431 livestock-associated *E*. *coli* genomes. Analysis of the 1,948 genomes identified 331 multilocus sequence types (STs), 44 clonal complexes (CCs), and 149 singletons (STs that did not share alleles at six out of seven loci with any other ST). Most STs were only found in one source type, with 192 human-specific STs (1,261/1,517 isolates [83%]), 98 livestock-specific STs (225/411 isolates [55%]), and 4 meat-specific STs (4/20 isolates [20%]). Thirty-five STs contained isolates from both humans and livestock/meat (*n* = 431), while 2 STs were found only in isolates from livestock and meat (*n* = 27) (Table S1). The three most common STs associated with bloodstream infection were ST73, ST131, and ST95, while the three most common STs associated with livestock were ST10, ST117, and ST602 (Table S1), although the distribution of STs varied depending on the livestock host (Table S2). The greatest overlap in STs between the two reservoirs occurred in ST10 (3%, 16%, and 0% of human, livestock, and meat isolates, respectively), and ST117 (1%, 8%, and 20% of human, livestock, and meat isolates, respectively). Phylogroups varied depending on the source, with B2 predominating in the human invasive samples (1,026/1,517 [68%]), but B2 was identified in only 4 livestock samples (pig = 2, turkey = 1, beef cattle = 1) (Table S2).

10.1128/mBio.02693-18.8 TABLE S2 Distribution of the five most frequent sequence types (STs) and each phylogroup per source. Download Table S2, PDF file, 0.1 MB. Copyright © 2019 Ludden et al. 2019 Ludden et al. This content is distributed under the terms of the Creative Commons Attribution 4.0 International license.

Phylogenies based on single nucleotide polymorphisms (SNPs) in the core (conserved) genomes of isolates representing CC10 (*n* = 149) and CC117 (*n* = 64) demonstrated that human isolates were intermixed with livestock isolates in CC10, but were generally distinct from livestock isolates in CC117 (Fig. S2 and S3). Pairwise SNP analysis demonstrated that the most closely related human/livestock isolate pairs were 85 and 96 SNPs different for CC10 and CC117, respectively. The estimated mutation rate for E. coli is one SNP/core genome/year (19, 20), and so CC10 and CC117 isolates in humans and livestock were not associated with recent transmission between the two groups. Combining the study CC117 isolates with 7 publicly available ST117 genomes (NCBI SRA accession numbers ERR769196, ERR769195, ERR769183, ERR769169, SRR1314275, SRR3410778, and SRR3438297) in a Bayesian phylogenetic analysis provided further evidence for the lack of recent transmission between human and livestock hosts in our study. The dated phylogeny revealed a UK cluster of 47 CC117 isolates (containing 44 turkey, 1 chicken, and 2 human isolates), for which the estimated time of most recent common ancestor (TMRCA) was 1989 (95% highest posterior density interval [HPD], 1979 to 1996), coinciding with the first global report of *bla*_CTX-M-1_ (21). Of the 47 isolates, 36 (77%) carried *bla*_CTX-M-1,_ which was uncommon in the rest of the bacterial population. All 36 isolates were from turkeys, representing a *bla*_CTX-M-1_ poultry-associated lineage, for which the TMRCA was 2011 (95% HPD, 2010 to 2013) (Fig. S4), suggesting acquisition of *bla*_CTX-M-1_ by this lineage between 1989 and 2011.

10.1128/mBio.02693-18.2 FIG S2 Maximum likelihood phylogenetic tree constructed from core gene SNPs identified for 149 *E. coli* isolates belonging to clonal complex 10, associated with bloodstream infection (*n* = 57), livestock (*n* = 90), retail meat (*n* = 1), and the *E. coli* MG1655 K-12 reference (*n* = 1). Download FIG S2, TIF file, 0.6 MB. Copyright © 2019 Ludden et al. 2019 Ludden et al. This content is distributed under the terms of the Creative Commons Attribution 4.0 International license.

10.1128/mBio.02693-18.3 FIG S3 Maximum likelihood phylogenetic tree constructed from core gene SNPs identified for 64 *E. coli* isolates belonging to clonal complex 117, associated with bloodstream infection (*n* = 12), livestock (*n* = 48), and retail meat (*n* = 5). Download FIG S3, TIF file, 0.4 MB. Copyright © 2019 Ludden et al. 2019 Ludden et al. This content is distributed under the terms of the Creative Commons Attribution 4.0 International license.

10.1128/mBio.02693-18.4 FIG S4 Dated Bayesian maximum likelihood phylogenetic tree of CC117 isolates from livestock, meat, and human invasive infections from the UK and 7 isolates from Denmark and the United States. Download FIG S4, TIF file, 0.4 MB. Copyright © 2019 Ludden et al. 2019 Ludden et al. This content is distributed under the terms of the Creative Commons Attribution 4.0 International license.

**FIG 2:**
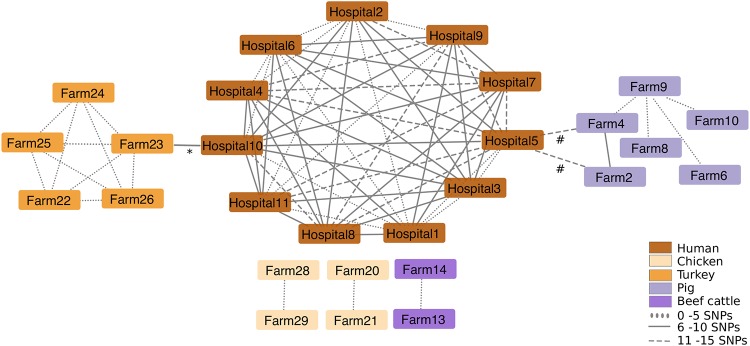
Network analysis of E. coli isolates cultured from livestock farms and patients with bloodstream infection. The results shown are limited to those isolate pairs identified in a pairwise comparison that differed by ≤15 or less SNPs in the core genome. The place of origin for each isolate pair are connected by lines, and the style of the line reflects the SNP distance. The asterisk indicates one ST69 human isolate from hospital 10 linked to two ST69 turkey isolates from farm 23 that differed by 10 and 12 SNPs, respectively. The number or hash sign indicates one ST1081 human isolate from hospital 5 linked to one ST1081 pig isolate from farm 4 (differed by 10 SNPs) and 2 ST1081 (probably duplicate) pig isolates from farm 2 that differed by 14 SNPs.

We then compared the genetic relatedness of the 431 livestock/meat E. coli isolates with the 1,517 E. coli isolates associated with human bloodstream infections. A maximum likelihood phylogenetic tree of the 1,948 genomes based on 277,533 core gene SNPs demonstrated high genetic diversity overall, with limited phylogenetic intermixing between isolates from humans and livestock (Fig. 1c). Pairwise SNP analysis between human- and livestock/meat-associated isolates demonstrated a median SNP distance of 41,658 (range, 10 to 47,819; interquartile range [IQR], 34,730 to 42,348), with 5 and 1 human isolates falling within 50 SNPs of livestock and meat, respectively (Fig. S5). Network analysis based on a range of SNP cutoffs captured just 2 (0.1%) human isolates (from hospitals in the South East and North West) that were within 15 SNPs of livestock isolates (2 pig isolates and 1 turkey isolate from three different farms [Fig. 2]). In contrast, we observed highly related isolates (0 to 5 SNPs) from the same animal species on different farms (Fig. 2).

10.1128/mBio.02693-18.5 FIG S5 Barplot of pairwise SNP differences between 1,928 isolates from humans (*n* = 1,517) and livestock (*n* = 411), with the frequency representing the number of human isolates related to a livestock isolate at the SNP threshold defined on the *x* axis. A range of SNPs from 0 to 500 is shown. Download FIG S5, TIF file, 0.5 MB. Copyright © 2019 Ludden et al. 2019 Ludden et al. This content is distributed under the terms of the Creative Commons Attribution 4.0 International license.

The E. coli isolated during this study are likely to be an underrepresentation of the diversity of E. coli in the wider UK livestock population and the meat sold in supermarkets, which could reduce our power to detect a transmission event between livestock and humans or vice versa. To explore this further, we undertook additional analyses using UK livestock isolates in the public domain; specifically, the published livestock genomes held in Enterobase (http://enterobase.warwick.ac.uk), which comprised 51 genomes of isolates cultured between 1999 and 2013. The 24 STs in this collection were compared with the STs assigned to our invasive isolate collection from across the UK. A single ST in this new data set was also present in our bloodstream collection (ST398), but this had already been identified in our livestock collection. This provides further support that sharing of invasive E. coli lineages between humans and livestock in the UK is uncommon.

We evaluated and compared the accessory (non-conserved) genome of the 1,948 study isolates using principal-component analysis (PCA). Principal components 1 (PC1) and 2 (PC2), which accounted for 50.5% and 8.3% of the variation within the data, respectively, separated the collection into two main clusters (referred to as group 1 or group 2, respectively). Group 1 predominantly contained human isolates, and group 2 contained a mixture of human and livestock isolates (Fig. S6a). PCA also showed that isolates from the same STs clustered together and formed distinct subclusters within groups 1 and 2 (Fig. S6b). Table S3 lists the top 100 genes from PC1 and PC2 that were most strongly associated with group 1 or 2.

10.1128/mBio.02693-18.6 FIG S6 Principal component analysis based on the presence/absence of accessory genes in 1948 *E. coli* isolates associated with bloodstream infection (*n* = 1517), livestock (*n* = 411), and retail meat (*n* = 20). (a) Principal component 1 (PC1) (*x* axis) against PC2 (*y* axis) labeled by source. (b) Principal component 1 (PC) (*x* axis) against PC2 (*y* axis) labeled by major sequence types (STs). Download FIG S6, TIF file, 0.6 MB. Copyright © 2019 Ludden et al. 2019 Ludden et al. This content is distributed under the terms of the Creative Commons Attribution 4.0 International license.

10.1128/mBio.02693-18.9 TABLE S3 The top 100 genes from PC1 and PC2 that are most strongly associated with either group 1 or group 2. Download Table S3, XLSX file, 0.01 MB. Copyright © 2019 Ludden et al. 2019 Ludden et al. This content is distributed under the terms of the Creative Commons Attribution 4.0 International license.

### Genetic analysis of antimicrobial resistance genes and associated mobile genetic elements.

**FIG 3:**
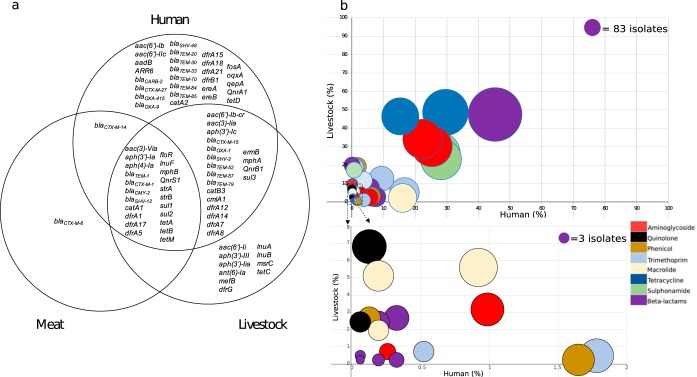
(a) Venn diagram displaying antibiotic resistance genes identified in 1,948 E. coli isolates cultured from livestock, meat, and patients with bloodstream infections. (b) Bubble graph showing the proportion of genes shared between E. coli from humans and livestock. The bottom graph shows an expanded view of very low prevalence genes that are clustered in the lower left-hand corner of the graph. The size of each bubble represents the number of isolates that the gene was identified in. Bubbles are colored by antibiotic class.

Screening of the 1,948 isolates for accessory genes encoding antibiotic resistance revealed that 41 different resistance genes were present in isolates from both humans and livestock (Fig. 3a). The prevalence of resistance genes in the two groups varied considerably, with some predominating in the human or livestock reservoir only, while others were common in both (Fig. 3b). The seven most frequently shared genes (each present in >300 isolates) conferred resistance to beta-lactams (*bla*_TEM-1_ = 882), sulfonamides (*sul2 *=* *530, *sul1 *=* *522), aminoglycosides (*strA *=* *509, *strB *=* *478), and tetracyclines (*tetA *=* *423, *tetB *=* *335). The predominant genes conferring resistance to extended-spectrum cephalosporins were *bla*_CTX-M-15_ (human = 87, livestock = 32) and *bla*_CTX-M-1_ (human = 1, livestock = 82, meat = 13). No carbapenemase or colistin resistance genes were detected.

**FIG 4:**
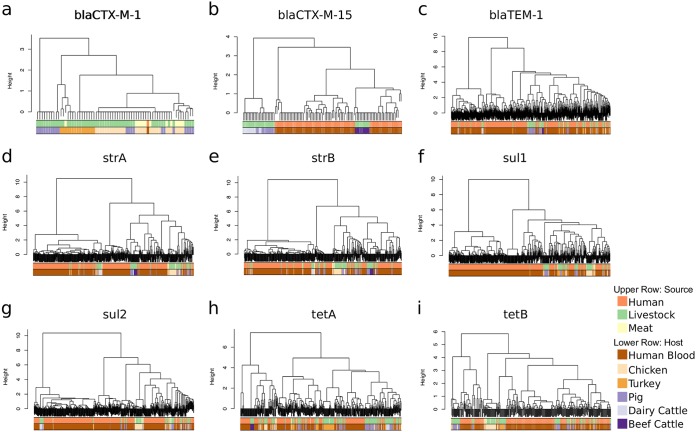
Dendrograms of mobile genetic element clusters identified for *bla*_CTX-M-1_ (a), *bla*_CTX-M-15_ (b), *bla*_TEM-1_ (c), *strA* (d), *strB* (e), *sul1* (f), *sul2* (g), *tetA* (h), and *tetB* (i) in livestock, humans, and retail meat.

To better understand whether genes encoding resistance in isolates from livestock and humans were carried by the same or different mobile genetic elements, contigs containing the specific gene of interest were extracted, and isolates were clustered using hierarchical cluster analysis based on contig presence/absence. This was performed for each of the seven most common resistance genes (*bla*_TEM-1,_ *sul2*, *sul1*, *strA*, *strB*, *tetA*, and *tetB*) and the two most prevalent ESBL genes (*bla*_CTX-M-15_ and *bla*_CTX-M-1_). Using a height cutoff value of 0 (corresponding to an identical mobile genetic element [MGE] contig carriage profile), clusters were screened for the presence of isolates derived from both human and livestock/meat. The majority of human isolates did not reside in clusters containing animal samples, the exception being *bla*_CTX-M-1_ where a single human isolate carrying this gene clustered with 23 animal isolates (Fig. 4). For the nine resistance genes analyzed, between 0.6% and 9.8% of human isolates carrying a resistance gene shared a cluster with livestock isolates. The lowest frequency of relatedness was observed for *bla*_TEM-1_ (3 clusters, involving isolates from humans = 4, livestock = 3), followed by *sul1* (3 clusters, humans = 3, livestock = 8), *strA* (6 clusters, humans = 11, livestock = 22), *tetA* (6 clusters, humans = 7, livestock = 19), *tetB* (5 clusters, humans = 11, livestock = 9), *bla*_CTX-M-15_ (1 cluster, humans = 5, livestock = 10), *sul2* (9 clusters, humans = 41, livestock = 38, meat = 1), *strB* (8 clusters, humans = 33, livestock = 29), and *bla*_CTX-M-1_ (1 cluster, humans = 1, livestock = 17, meat = 6) (Table S4). Individual clusters often contained different STs (Table S4), which is indicative of horizontal transfer of mobile genetic elements between lineages.

10.1128/mBio.02693-18.10 TABLE S4 Details of common mobile elements (denoted as “profiles”) identified in *E. coli* associated with bloodstream infection, livestock, and retail meat for *bla*_CTX-M-1_, *bla*_CTX-M-15_, *bla*_TEM-1_, *strA*, *strB*, *sul1*, *sul2*, *tetA*, and *tetB*. Download Table S4, XLSX file, 0.1 MB. Copyright © 2019 Ludden et al. 2019 Ludden et al. This content is distributed under the terms of the Creative Commons Attribution 4.0 International license.

Further characterization of *bla*_CTX-M_ plasmids was undertaken using long-read sequencing. Two livestock-human isolate pairs positive for *bla*_CTX-M-1_ or *bla*_CTX-M-15_ were selected for sequencing using the PacBio RSII instrument. Illumina reads for the entire study collection were then mapped to the complete plasmid assemblies of these four isolates. The single *bla*_CTX-M-1_-positive human isolate contained an IncI1 *bla*_CTX-M-1_ plasmid that was highly similar (>99% identity and ≥98% coverage) to 28 livestock isolates (chicken = 18, chicken meat = 8, pig = 2) belonging to four different STs. In contrast, the *bla*_CTX-M-15_ plasmid in the livestock (E01) and human (D01) isolate pair were dissimilar (17% sequence shared at 99% identity [ID]) and had different replicon types (E01 = IncHI2, D01 = IncFIA and FII fusion). The human *bla*_CTX-M-15_ plasmid (D01) was not identified in any other isolate (human or livestock), while the livestock *bla*_CTX-M-15_ plasmid (E01) was found in other livestock isolates from the same pooled fecal sample from 1 beef farm.

We then investigated whether *bla*_CTX-M-15_ could be shared on a smaller transposable element. A 7,926-bp region encoding *bla*_CTX-M-15_ that was identical to a Tn*3* transposon previously identified from E. coli plasmid GU371928 (22) was detected in 22/32 (69%) livestock isolates (pig = 11, dairy cattle = 11) from 4 farms, and 3/87 (3%) of *bla*_CTX-M-15_-positive human isolates from 2 hospitals, one of which was located in the East of England. This 7,926-bp region was flanked by 5-bp direct repeats of TTTTA, indicating its potential for transfer between isolates.

## DISCUSSION

We investigated the prevalence and genetic relatedness of E. coli from livestock, meat, and humans in the East of England using a “One Health” approach. ESBL-E. coli was isolated from 55% of livestock farms, with a frequency of ESBL-E. coli in different livestock species that was consistent with previous findings (23). In addition, ESBL-E. coli bacteria were found in 18% of prepackaged fresh meat products. The high prevalence of ESBL-E. coli in chicken meat (16/30 [53%]) is similar to previous studies conducted in the UK and the Netherlands (12, 24, 25). However, E. coli from livestock were not closely related to isolates causing human disease in our region, suggesting that livestock are not a direct source of infecting isolates and that human invasive E. coli are not being shared with livestock. E. coli phylogroup B2 was most frequently associated with human invasive samples (68%) as previously reported (26), but was rarely identified in livestock (1%), providing further evidence for distinct populations associated with invasive human disease and livestock. In contrast, highly related isolates were identified between the same livestock species on different farms. Previous studies in the Netherlands that compared isolates from clinical and livestock sources using MLST indicated that the same ST could be isolated from humans and livestock (12–14, 27). We replicated this finding for CC10 and CC117, but using the more discriminatory sequence-based analysis identified that isolates from the two reservoirs were genetically distinct. A study of cephalosporin-resistant E. coli in the Netherlands (15) reported genetic heterogeneity between human and poultry-associated isolates but closely related isolates from farmers and their pigs. Here, we included ESBL-positive and non-ESBL E. coli, an important feature of the study since the majority of E. coli human infections in the UK are due to non-ESBL E. coli (17).

Screening of E. coli isolates from livestock, meat, and humans with serious infections revealed the frequency of antimicrobial-resistant genes in each reservoir and confirmed the presence of similar antimicrobial resistance genes in both livestock and humans, including *bla*_TEM-1_, *sul2*, *sul1*, *strA*, *strB*, *tetA*, *tetB, bla*_CTX-M-15_, and *bla*_CTX-M-1_. These genes confer resistance to four antibiotic classes, all of which are used in both livestock and humans (28). This confirms their ubiquitous distribution but does not provide evidence for recent transfer of genes between the two reservoirs. To address this, we hypothesized that recent sharing would be associated with transmission via the same or highly related mobile genetic elements (MGEs), as previously suggested for ESBL genes (15). Previous studies have highlighted the challenge in reconstructing plasmids and other mobile elements encoding resistance genes from whole-genome sequencing (29, 30), hindering our understanding of the transmission dynamics of resistance genes. We developed an approach to detect and genetically compare mobile elements across our large study collection, with validation of findings for ESBLs using long-read sequencing. The findings from this were consistent with predominantly distinct mobile elements between livestock and humans, with an estimated 69/1,517 (5%) human isolates potentially sharing closely related antimicrobial resistance-associated mobile elements with those found in livestock.

Our study has several limitations. We acknowledge that the E. coli from humans predated the surveys of farms and retail meat but took account of this by identifying relatedness based on a 0 to 15 SNP cutoff given the estimated E. coli mutation rate of 1 SNP/core genome/year (19, 20). We did not include all possible sources of E. coli for humans (for example, vegetables, fruits, and pets), although a recent study found no E. coli with *bla*_CTX-M-15_ (the dominant human ESBL type) in retail meat, fruit, and vegetables in five UK regions (24). Additional studies are required to understand whether our findings will be reproduced in other geographical areas, to determine other sources of invasive lineages such as wastewater or recreational waters, to better understand within-host diversity in livestock, to differentiate between historical and recent transmission events by collecting data over a longer time period, and to identify whether livestock are a source for other types of infection in humans such as urinary tract infections.

In conclusion, this study has not generated evidence to indicate that E. coli causing severe human infections in our region were derived recently from livestock, with host-specific E. coli lineages identified from hospitals versus farms. We identified limited sharing of antimicrobial resistance genes between livestock and humans based on long-read sequencing and analysis of mobile genetic elements. Further investigations are required to pursue the identification of the source of E. coli and resistance genes in isolates associated with severe human disease.

## MATERIALS AND METHODS

### Sampling of livestock feces and retail meat.

A cross-sectional survey was performed between August 2014 and April 2015 to isolate E. coli at 20 livestock farms (10 cattle and 10 pig) in the East of England. A pooled sample of approximately 50 g of freshly passed fecal material was collected from each major area in a given farm (such as different pens) using a sterile scoop (Sterilin X400; Thermo Fisher Scientific, Loughborough, United Kingdom). Each pooled sample was placed into a dry sterile 150-ml container (Sterilin polystyrene containers; Fisher Scientific). A median of 4 samples (range, 1 to 5) were taken from each cattle farm, and a median of 4.5 samples (range, 3 to 9) were taken from each pig farm, resulting in a total of 85 pooled samples (34 cattle and 51 pig). In addition, cecal contents were collected from 2 deceased pigs at the time of necropsy.

Poultry reared at nine farms (4 chicken and 5 turkey) in the East of England were sampled at two abattoirs between February and April 2015. Two sample types were taken for each farm: (i) pooled feces with a total weight of approximately 50 g from 10 to 20 transportation crates immediately after the livestock were removed; (ii) pools of cecal material from up to 10 birds after slaughter. Each sample was taken using a sterile scoop, and a sterile surgical scalpel was used for each cecal dissection. A median of 4 (range, 2 to 4) cecal pools and 4 (range, 3 to 4) fecal pools were collected from livestock from each chicken farm, and a median of 1.5 (range, 1 to 2) cecal pools and 2.5 (range, 2 to 3) fecal pools were collected from livestock from each turkey farm. This resulted in a total of 49 pooled samples (29 chicken and 20 turkey). All samples were immediately refrigerated at 4°C upon return to the laboratory and processed on the same day.

In April 2015, 97 retail meat samples (beef [15], chicken [30], pork [42], turkey [7], venison [1], veal [1], mixed minced pork and beef [1]) were purchased from 11 supermarkets in Cambridge, UK, with 5 to 16 meat products collected from each supermarket that were selected to capture diversity in the products available. The country of origin for each meat product was recorded, and where multiple countries/regions were stated on the packaging, all names were recorded.

### Microbiology.

Pooled fecal samples were diluted 1:1 with sterile phosphate-buffered saline and mixed vigorously, and 100-μl aliquots were plated onto Chromocult coliform agar (VWR, Leuven, Belgium) and *Brilliance* ESBL agar (Oxoid, Basingstoke, UK), which are selective chromogenic agars that support the growth of coliforms and ESBL-producing organisms, respectively. Agar plates were incubated at 37°C for 48 h in air prior to inspection. Enrichment cultures were also used to detect ESBL-producing E. coli by adding 1 ml of fecal preparation to 9 ml of tryptic soy broth containing 20 µg cefpodoxime and incubating for 24 h in a shaking incubator (150 rpm) at 37°C in air, before 100 μl was plated onto *Brilliance* ESBL agar and incubated for 48 h in air. Numerous E. coli colonies were picked from primary cultures of positive pooled stool samples based on diversity in colonial morphology. Up to 32 colonies of presumptive E. coli based on colony morphology were picked from samples taken on each farm.

Preparation and culture of meat samples followed the European standard ISO 6887–2:2003. All exterior packaging was disinfected with alcohol prior to removal of meat. A 5-g sample of meat was aseptically removed, added to 45 ml peptone broth, and homogenized using a Stomacher paddle blender (Stomacher80 Laboratory System, Seward Ltd., UK) for 2 min. Samples were transferred into 50-ml Falcon tubes and incubated in a shaking incubator for 24 h at 150 rpm at 37°C. After incubation, all samples were plated onto *Brilliance* ESBL agar and incubated at 37°C for 48 h. In addition, swabs were obtained from whole chicken carcasses and incubated in 3 ml brain heart infusion (BHI) broth (FlOQSwabs; Copan Italia spa, Brescia, Italy) in a shaking incubator for 24 h at 150 rpm at 37°C. Following incubation, 100 μl was plated onto *Brilliance* ESBL agar and incubated as described before. One colony of presumptive ESBL E. coli was picked from each *Brilliance* ESBL agar for further evaluation, with the exception of two meat samples, where two colonies were picked to represent each of two distinct colony morphologies.

All bacterial colonies suspected to be E. coli were identified to the species level using matrix-assisted laser desorption ionization–time of flight mass spectrometry (Bruker Daltonik, Bremen, Germany). Antimicrobial susceptibility was defined for each E. coli colony using the Vitek2 system (bioMérieux, Marcy l’Etoile, France) with the AST-N206 card and calibrated against EUCAST breakpoints (http://www.eucast.org/clinical_breakpoints/).

### DNA sequencing.

Bacterial genomic DNA was extracted using the QIAxtractor (Qiagen, Valencia, CA, USA) according to the manufacturer's instructions. Library preparation was conducted according to the Illumina protocol and sequenced on an Illumina HiSeq2000 (Illumina, San Diego, CA, USA) with 100-cycle paired-end runs. Sequence data were retrieved for a further 1,517 open access E. coli isolates associated with bloodstream infections (17, 18). Of these, 424 were isolated between January 2006 and December 2012 at the Cambridge University Hospitals NHS Foundation Trust, and 1,093 were submitted to the British Society for Antimicrobial Chemotherapy Bacteraemia Resistance Surveillance Project by 11 UK hospitals between 2001 and 2011 (for details, see www. bsacsurv.org and Table S1 in the supplemental material) (31). Previous description and analysis of these genomes (17, 18) did not include comparisons with isolates from livestock or meat.

### Genome assembly, annotation, and multilocus sequence typing.

Taxonomic identity was assigned using Kraken (32). One isolate (VREC0294) was found to not be E. coli and was excluded from further analysis. Multiple assemblies were created using VelvetOptimiser v2.2.5 (33) and Velvet v1.2 (34). An assembly improvement step was applied to the assembly with the best N50, contigs were scaffolded using SSPACE (35), and sequence gaps were filled using GapFiller (36). Assemblies were annotated using Prokka v1.5 (37), and genus-specific databases from RefSeq (38). Multilocus sequence types (MLSTs) were identified using the MLST sequence archive (https://enterobase.warwick.ac.uk). Sequence types (STs) were classified into clonal complexes using the eBURST V3 algorithm (http://eburst.mlst.net/). *In silico* E. coli phylotyping was performed using ClermonTyping (39).

### Pan-genome analysis.

The pan-genome was calculated for all 1,948 isolates using Roary (40), with a 90% ID cutoff and genes classified as “core” if they were present in at least 99% of isolates. A maximum likelihood tree was created using RAxML (41) based on single nucleotide polymorphisms (SNPs) in the core genes. Principal-component analysis was performed across the 1,948 isolates based on the accessory genes from Roary using R. A Spearman rho correlation analysis was performed on the principal components, the gene absence/presence data, and the ST and source of isolation metadata.

### Phylogeny-based analysis of individual lineages.

Lineage-specific analyses were performed by mapping the sequence reads for isolates belonging to clonal complex 10 (CC10) and CC117 to an E. coli reference genome from the same clonal complex using SMALT 0.7.4 (http://www.sanger.ac.uk/resources/software/smalt/). *E*. *coli* MG1655 K-12 (ENA accession number U00096.2) was used as the reference genome for CC10, and a *de novo* assembly of the CC117 study isolate (ENA accession number ERR1204146) with the lowest number of contigs was used as the reference genome for CC117 as no reference genomes were available. To create a “core” genome, mobile genetic elements (MGEs) were identified using gene annotation, PHAST (phast.wishartlab.com), and BLAST (https://blast.ncbi.nlm.nih.gov) and removed, together with contigs less than 500 bp in length. Recombination was removed using Gubbins (42). A maximum likelihood phylogeny was created using RAxML (41) with 100 bootstraps and a midpoint root. Genetic diversity was calculated based on pairwise differences in SNPs in the core genomes using an in-house script. Visualization of phylogenetic trees was performed using iToL (http://itol.embl.de) (43) and FigTree v 1.4.2 (http://tree.bio.ed.ac.uk/software/figtree/).

### Bayesian Evolutionary Analysis Sampling Trees (BEAST).

All published genomes for CC117 in the *Enterobase* online database (https://enterobase.warwick.ac.uk, accessed 10 June 2016) that had been generated on an Illumina instrument and had the country, year, and source of isolation available were identified (ERR769196, ERR769195, ERR769183, ERR769169, SRR1314275, SRR3410778, and SRR3438297). These were mapped to the CC117 reference using SMALT, combined with the CC117 study isolates, and mobile elements and recombination were removed as before. Dating of this lineage was completed using BEAST v.1-8 (44). BEAST v.1-8 was run using the Hasegawa, Kishino, and Yano (HKY) and gamma substitution model. We compared combinations of three population size change models (constant, exponential, and Bayesian skyline plot) and three molecular clock models (strict, exponential, and uncorrelated lognormal). A Bayesian skyline population model and an uncorrelated lognormal molecular clock were selected based on Bayes factors calculated from path sampling and stepping stone sampling (44, 45).

### Detection of antimicrobial resistance and mobile elements.

Acquired genes encoding antibiotic resistance were identified using Antibiotic Resistance Identification By Assembly (ARIBA), comparing the study genomes against an in-house curated version of the Resfinder database (46–48) consisting of 2,015 known resistance gene variants. Genes were classified as present using an identity of 90% nucleotide similarity. Genes reported as fragmented, partial, or interrupted were excluded.

For all isolates positive for the *bla*_CTX-M-1_, *bla*_CTX-M-15_, *bla*_TEM-1_, *sul1*, *strA*, *strB*, *sul2*, *tetA*, and *tetB* genes, whole-genome assemblies were screened to identify the contig carrying the antimicrobial resistance (AMR) gene using the blastn application (49) with the AMR gene sequence as the query sequence. The identified contigs were then aligned against a previously curated database of complete Enterobacteriaceae plasmids (50) in order to filter out sequences representing E. coli chromosome fragments. For each AMR gene, a database containing unique AMR-carrying contigs was created using cd-hit-est (51) based on 90% identity cutoff. To determine contig carriage, for each of the eight AMR genes, all isolates positive for that gene were mapped against the respective gene-specific database of contigs using short-read sequencing typing (SRST2) using a minimum 90% coverage cutoff. The contig presence/absence data were converted into a distance matrix, and hierarchical clustering was performed using R function *hclust* and the ward.D2 method.

To examine plasmids carrying the ESBL genes *bla*_CTX-M-15_ and *bla*_CTX-M-1_, two pairs of livestock and human isolates were selected for sequencing on the PacBio RSII instrument (Pacific Biosciences, Menlo Park, CA, USA) (*n* = 4), and *in silico* PCR was used to perform plasmid incompatibility group/replicon typing (52). These two genes were selected, as they were the most prevalent ESBL genes found in the 1,948 isolates. The pair of *bla*_CTX-M-15_-positive isolates was selected, as they contained 5 identical genes encoding antimicrobial resistance. The single human *bla*_CTX-M-1_-positive isolate in the collection was selected, and a livestock isolate with the most similar resistance gene profile was selected, with both isolates containing 3 identical genes encoding antimicrobial resistance. DNA was extracted using the phenol-chloroform method (53) and sequenced using the PacBio RS II instrument. Sequence reads were assembled *de novo* with HGAP v3 (54) within the SMRT Analysis version 2.3.0 software (https://www.pacb.com/products-and-services/analytical-software/smrt-analysis/ ), circularized using Circlator v1.1.3 (55) and Minimus 2 (56), and polished using the PacBio RS_Resequencing protocol and Quiver v1 (54). Fully assembled plasmids were compared using WebACT (http://www.webact.org) and BLASTn (https://blast.ncbi.nlm.nih.gov).

### Ethical approval.

The study protocol was approved by the Cambridge University Hospitals NHS Foundation Trust Research and Development Department (reference A093285) and the National Research Ethics Service East of England Ethics Committee (reference 12/EE/0439 and 14/EE/1123).

### Data availability.

Sequence data for all isolates have been submitted to the European Nucleotide Archive (www.ebi.ac.uk/ena) under study accession number PRJEB4681 (all human E. coli), PRJEB8774 (non-ESBL-producing E. coli from livestock), and PRJEB8776 (ESBL-producing E. coli from livestock and meat), with the accession numbers for individual isolates listed in Table S1 in the supplemental material.
