# Supplementary figures and images for: One Health Genomic Surveillance of Escherichia coli Demonstrates Distinct Lineages and Mobile Genetic Elements in Isolates from Humans versus Livestock
Source: mBio. 2019 Jan 22;10(1):e02693-18. doi: 10.1128/mBio.02693-18 (PMC6343043; doi:10.1128/mBio.02693-18)

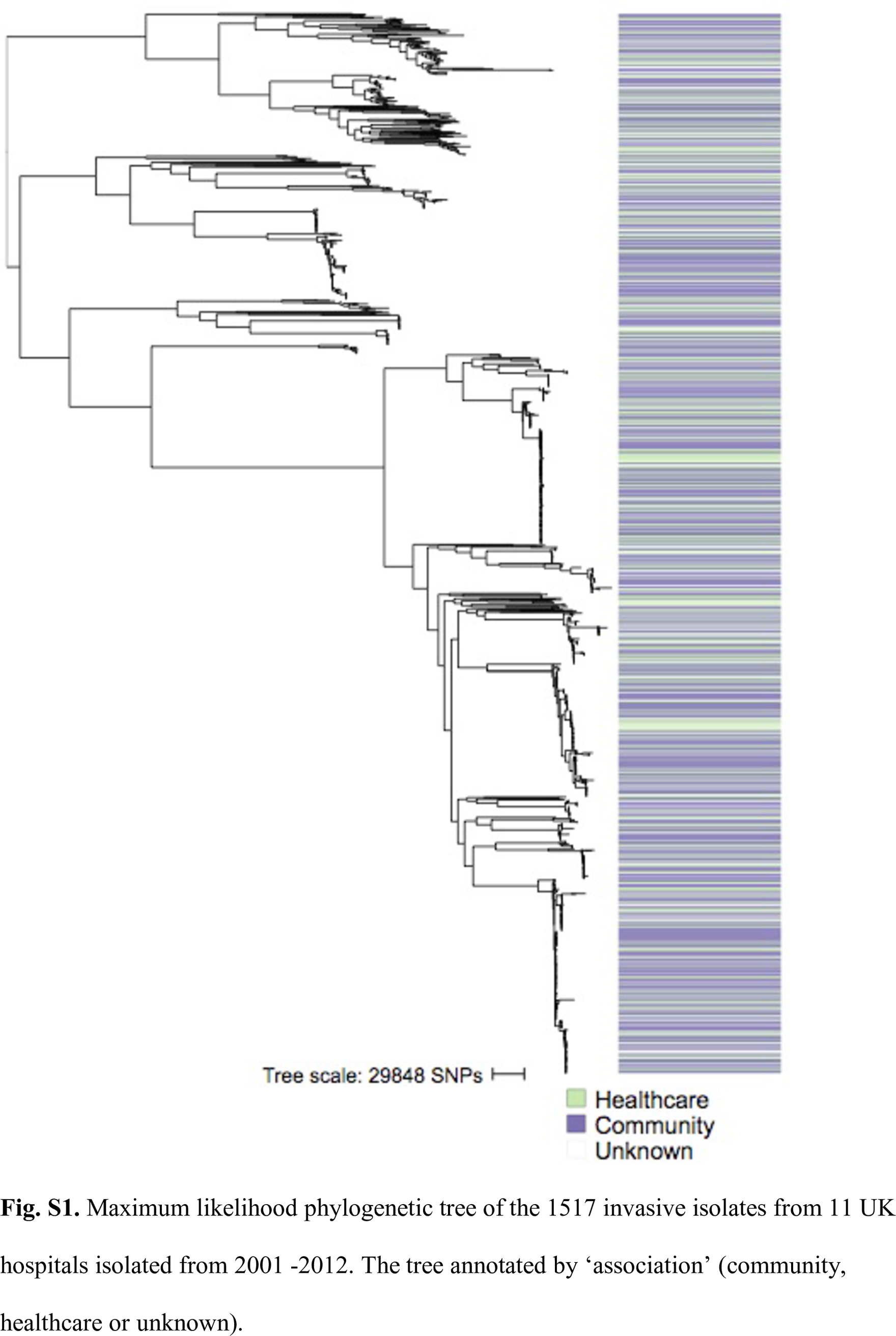

Supplement: FIG S1 [file mBio.02693-18-sf001.tif]

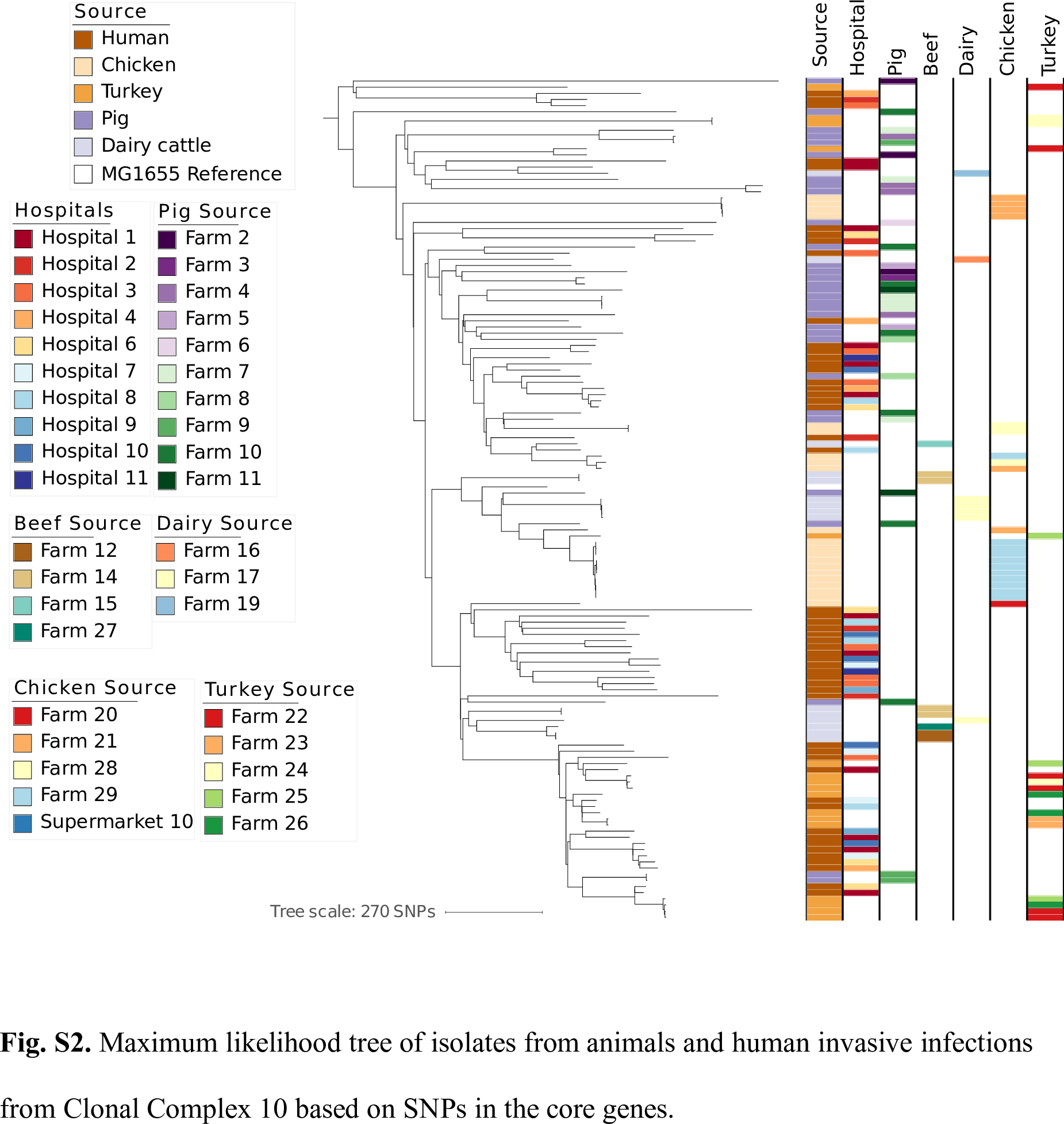

Supplement: FIG S2 [file mBio.02693-18-sf002.tif]

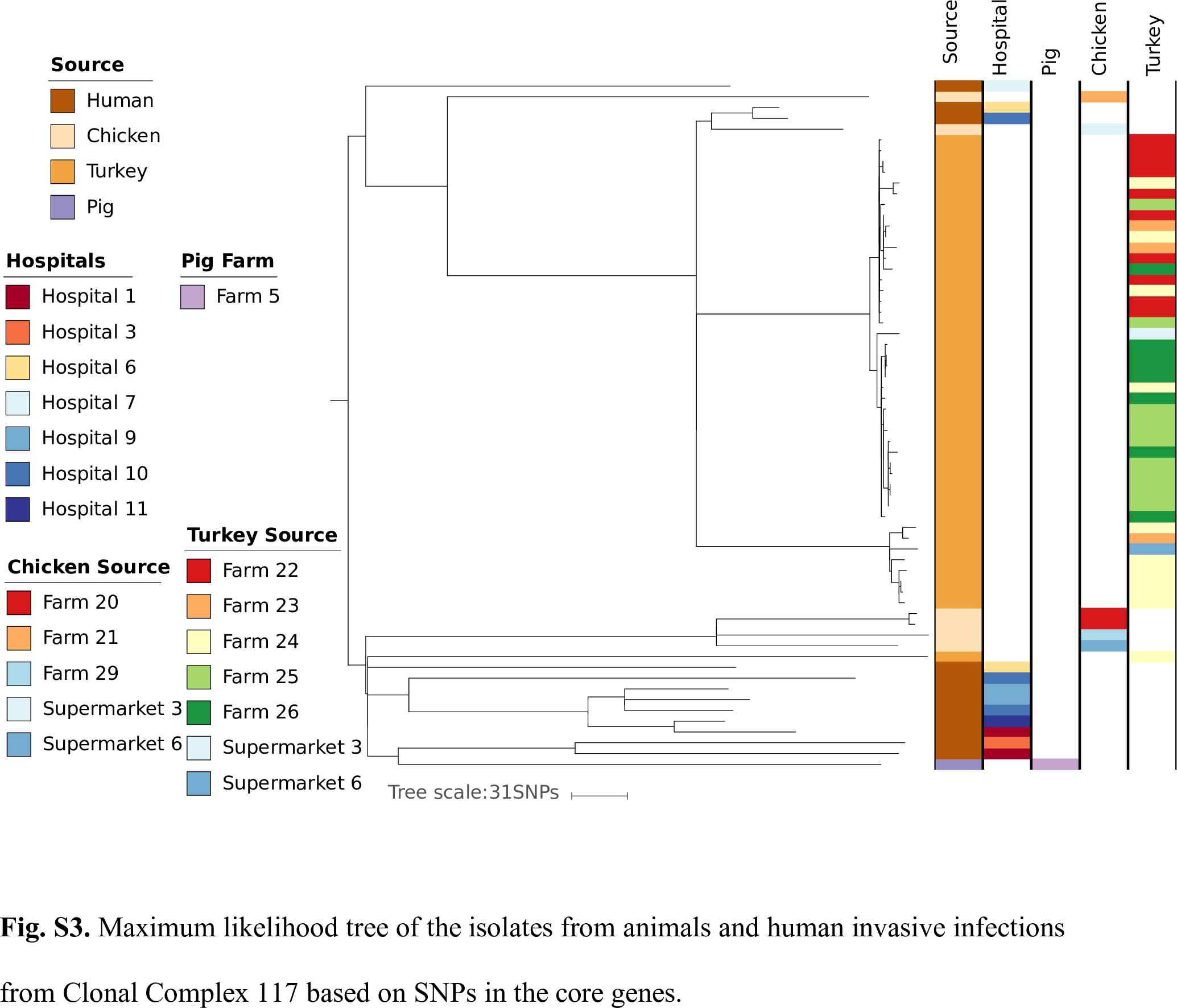

Supplement: FIG S3 [file mBio.02693-18-sf003.tif]

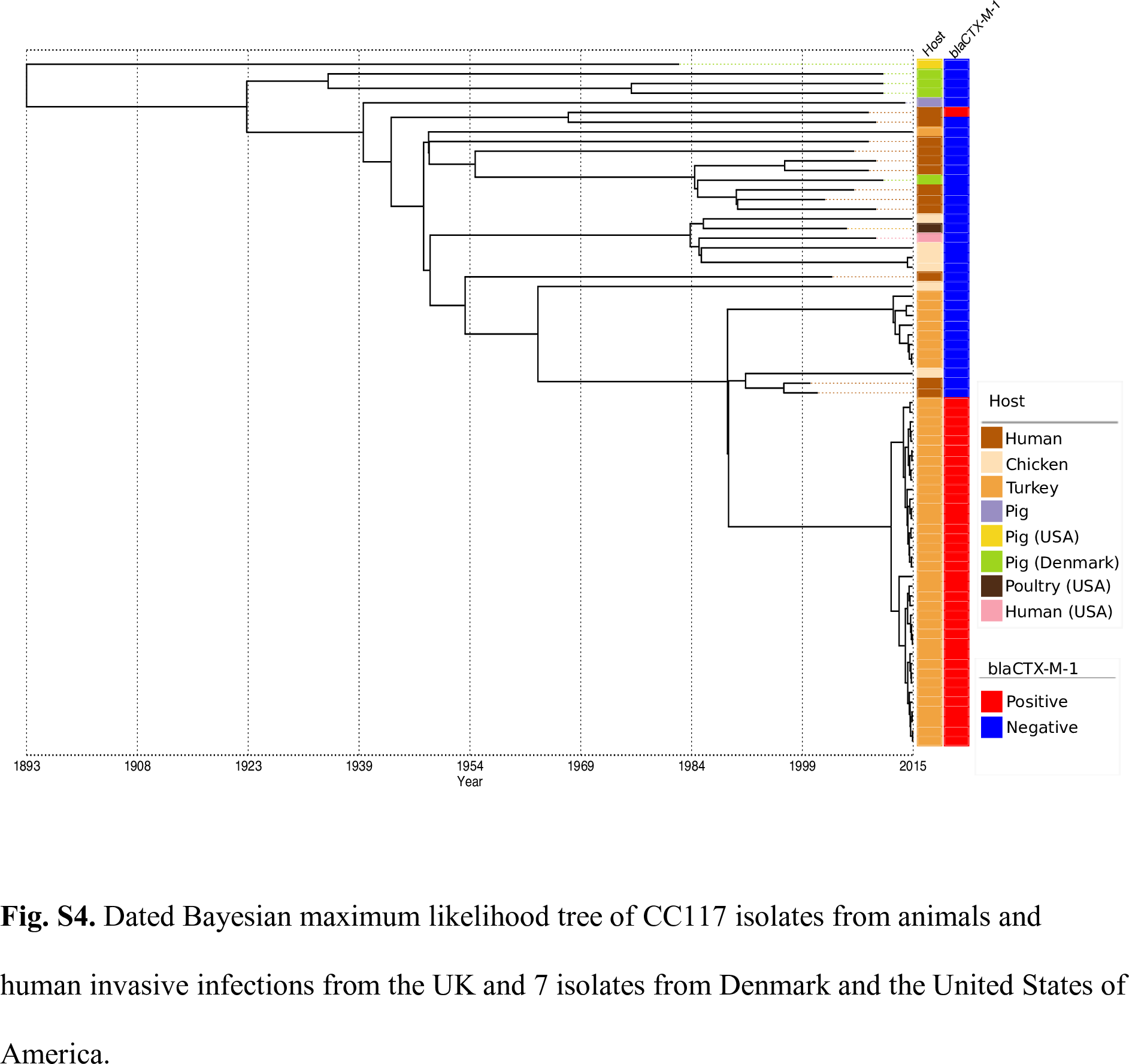

Supplement: FIG S4 [file mBio.02693-18-sf004.tif]

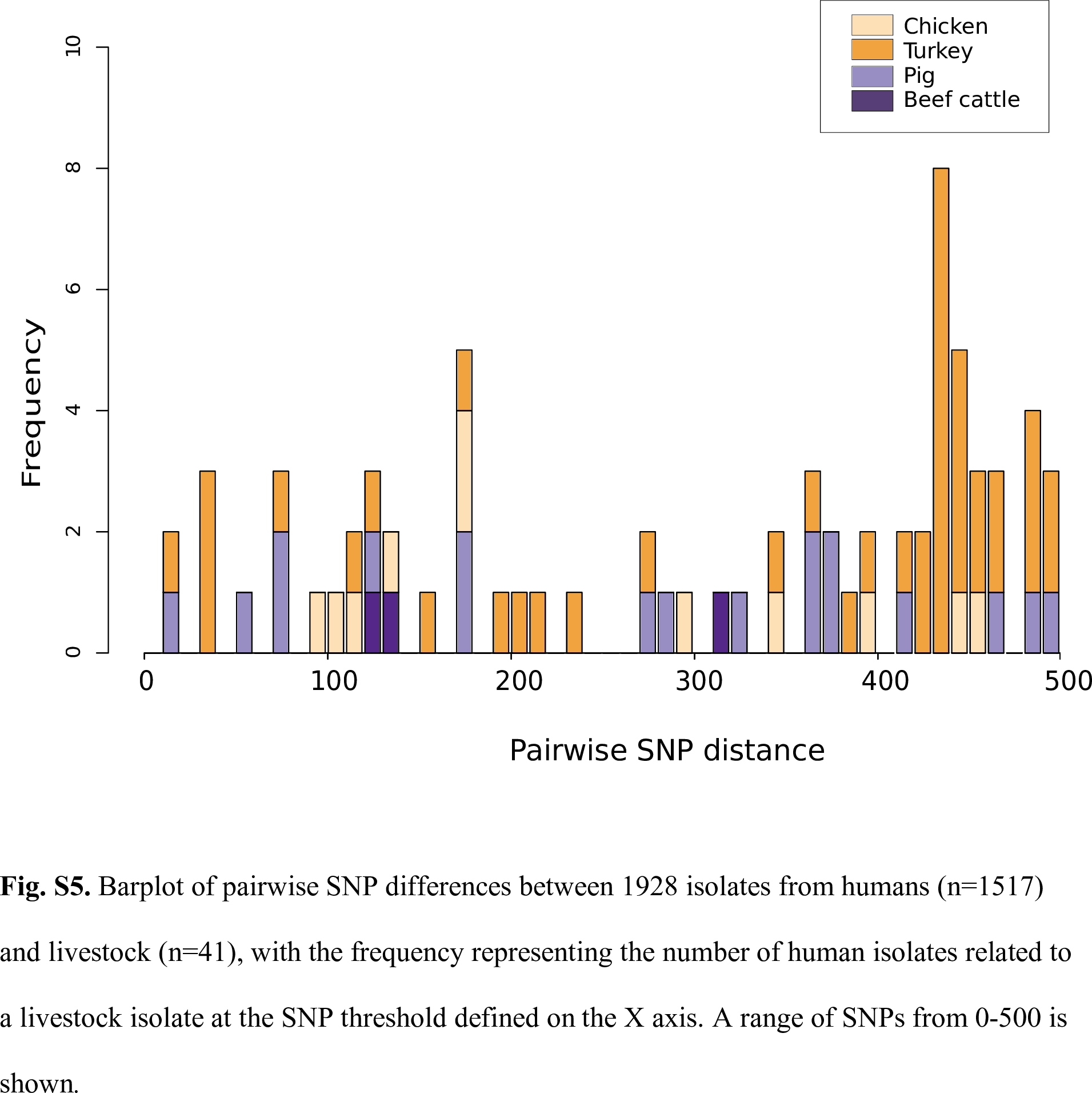

Supplement: FIG S5 [file mBio.02693-18-sf005.tif]

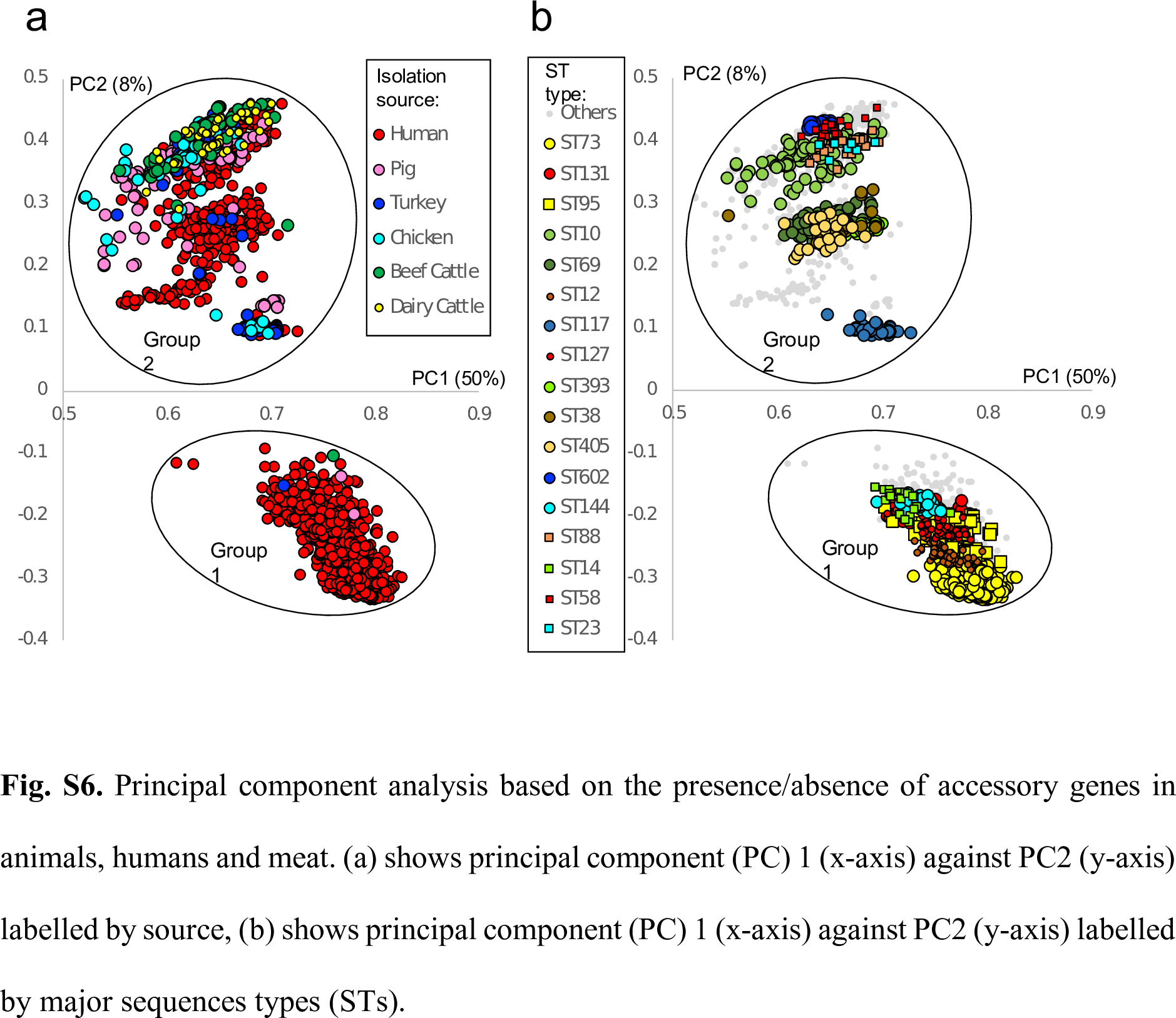

Supplement: FIG S6 [file mBio.02693-18-sf006.tif]
