# Supplementary material for: One Health Genomic Surveillance of Escherichia coli Demonstrates Distinct Lineages and Mobile Genetic Elements in Isolates from Humans versus Livestock
Source: mBio. 2019 Jan 22;10(1):e02693-18. doi: 10.1128/mBio.02693-18 (PMC6343043; doi:10.1128/mBio.02693-18)
Supplement: TABLE S2 [file mBio.02693-18-st002.pdf]

Supplementary Table 2 Distribution by source of the 5 most frequent sequence types (STs), and phylogroup

	Number (%) of isolates						Total (n=1948)
**ST**	Human (n=1517)	Chicken (n=84)	Turkey (n=102)	Dairy cattle (n=58)	Beef cattle (n=59)	Pig (n=128)	
ST 73	265 (17)	0	0	0	0	0	265 (14)
ST 602	0	24 (29)	0	0	0	0	24 (1)
ST 117	12 (0.8)	6 (7)	28 (27)	0	0	1 (0.8)	47 (2)
ST 2325	0	0	0	9 (16)	0	3 (2)	12 (0.6)
ST 196	0	0	0	0	10 (17)	0	10 (0.5)
ST 10	40 (3)	17 (20)	10 (10)	7 (12)	6 (10)	25 (20)	105 (5)
**Phylogroup**							
A	87 (6)	40 (48)	34 (33)	21 (36)	9 (15)	55 (43)	246 (13)
B1	69 (5)	31 (37)	13 (13)	36 (62)	47 (80)	30 (23)	226 (12)
B2	1026 (68)	0	1 (1)	0	1 (2)	2 (2)	1030 (53)
C	44 (3)	1 (1)	0	0	0	10 (8)	55 (3)
D	206 (14)	2 (2)	5 (5)	0	1 (2)	8 (6)	222 (11)
E	4 (0.3)	4 (5)	0	1 (2)	1 (2)	6 (5)	16 (0.8)
F	80 (5)	6 (7)	49 (48)	0	0	17 (13)	152 (8)
Unknown	1 (0.07)	0	0	0	0	0	1 (0.1)

Percentages are rounded to the nearest decimal
